# Associations of Adiponectin and Leptin with Incident Coronary Heart Disease and Ischemic Stroke in African Americans: The Jackson Heart Study

**DOI:** 10.3389/fpubh.2013.00016

**Published:** 2013-05-24

**Authors:** Aurelian Bidulescu, Jiankang Liu, Zhimin Chen, DeMarc A. Hickson, Solomon K. Musani, Tandaw E. Samdarshi, Ervin R. Fox, Herman A. Taylor, Gary H. Gibbons

**Affiliations:** ^1^Department of Community Health and Preventive Medicine, Morehouse School of MedicineAtlanta, GA, USA; ^2^Department of Medicine, University of Mississippi Medical CenterJackson, MS, USA; ^3^Rural Health Research Center, University of South CarolinaColumbia, SC, USA; ^4^School of Health Sciences, Jackson State UniversityJackson, MS, USA; ^5^National Heart Lung and Blood Institute, National Institutes of HealthBethesda, MD, USA

**Keywords:** cohort study, adiponectin, leptin, cardiovascular events, stroke, minorities, African Americans

## Abstract

**Background:** Because the predictive significance of previously reported racial differences in leptin and adiponectin levels remains unclear, we assessed the prospective association of these adipokines with the risk of cardiovascular disease (CVD) events in African Americans, a population with a high prevalence of cardiometabolic risk factors.

**Methods:** Serum specimens from 4,571 Jackson Heart Study participants without prevalent CVD at baseline examination (2000–2004) were analyzed for adiponectin and leptin levels. Cox proportional hazard regression models were used to estimate the associations of the two adipokines with incident coronary heart disease (CHD) and incident ischemic stroke.

**Results:** During 6.2 years average of follow-up, 98 incident CHD and 87 incident ischemic stroke events were documented. Among study participants (64% women; mean age 54 ± 13 years), the mean (standard deviation, SD) was 6.04 (4.32) μg/mL in women and 4.03 (3.14) μg/mL in men for adiponectin and 37.35 (23.90) ng/mL in women and 11.03 (10.05) ng/mL in men for leptin. After multivariable adjustment that included age, body mass index, high-density lipoprotein cholesterol, triglycerides, C-reactive protein, insulin resistance by homeostasis model assessment for insulin resistance, systolic blood pressure, hypertension medication, smoking, and physical activity, adiponectin was directly associated in women with incident stroke, HR = 1.41 (1.04–1.91) per one SD increase (*p* = 0.03), but not in men (*p* = 0.42). It was not associated with incident CHD in women or men. Leptin was not associated with incident CHD or incident stroke.

**Conclusion:** In the largest community-based African American cohort, adiponectin was associated among women with a higher risk of incident stroke. Whether adiponectin harbors harmful properties, or it is produced in response to vascular inflammation to counter the atherosclerotic process, or the putative “adiponectin resistance” phenomenon acts, should be further assessed.

## Introduction

Significant research has been conducted in order to understand the obesity mechanisms related to cardiovascular disease (CVD) pathogenesis (1). It is now evident that adipose tissue is more than an energy storage compartment; it is a secretory organ for bioactive molecules known as adipokines (2). Adipokines such as leptin and adiponectin contribute to the pathophysiology of obesity-related disorders through their properties to modulate inflammatory and metabolic processes. In population studies leptin is directly associated with coronary heart disease (CHD) and ischemic stroke (3–7), but leptin’s proatherogenic properties in animal models are not clear (8, 9). Opposite to leptin, adiponectin is atheroprotective in animal models but epidemiological investigations evaluating its role in CVD have been contradictory (10, 11). Thus, given the conflicting studies in animal and human research and the reported racial differences in adipokine levels (12–14), we assessed the prospective association of the two adipokines with the risk of CVD events in African American participants in the Jackson Heart Study (JHS). Our study hypotheses were that there is a direct association between serum leptin levels and incident CVD (CHD and stroke) and an inverse association between serum adiponectin levels and incident CVD.

Because previous studies indicated gender-differences in the association of leptin and adiponectin with CVD (5, 15, 16), we also assessed if gender modifies the association of these adipokines with CVD events. Our *a priori* hypothesis was that there are differences by gender for these associations. We also assessed the effect modification by age groups, as previous studies reported a direct association between adiponectin and incident CHD among older individuals (17).

## Materials and Methods

### Study population

The JHS is a single-site, prospective cohort study of the risk factors and causes of heart disease in adult African Americans. A probability sample of 5,301 African Americans, aged 21–94 years, residing in a three county area surrounding the city of Jackson, MS, USA were recruited and examined at baseline (2000–2004) by certified technicians according to standardized protocols. Clinic visits and interviews occur approximately every 4 years. Annual follow-up interviews and cohort surveillance are ongoing. An overview of the JHS (18) and details of the study design (19), recruitment protocol (20), and data collection methods (19) are published elsewhere. The present study included 4,571 participants who had plasma leptin (*n* = 4,552) or plasma adiponectin (*n* = 4,554) measured using frozen specimens stored at JHS Exam 1 (2000–2004). Written consent was obtained from each participant at the inception of the study, and the study was approved by the participating JHS institutions: Jackson State University, Tougaloo College, and the University of Mississippi Medical Center. The study protocol for the ascertainment of the leptin and adiponectin samples was approved by the Morehouse School of Medicine Institutional Review Board.

### Ascertainment and adjudication of cardiovascular diseases incident events

Trained interviewers conduct telephone annual follow-up interviews to ascertain any significant health events since the last JHS contact, including diagnostic tests, hospitalizations, or death. Information on cohort hospitalizations and deaths is transmitted to the medical record abstraction unit who review death certificates and hospital records to identify CVD events in the cohort. Interviews with the next of kin and completed questionnaires by physicians and medical examiners or coroners are used to obtain information on deaths in the cohort. A computer-generated diagnosis with follow-up review and adjudication by trained medical personnel completes final, disease-specific event classification of hospitalized and fatal CHD and stroke (21).

### Leptin and adiponectin measurements

Venous blood samples were withdrawn from each subject at baseline examination after more than 8 h of fasting as described elsewhere (19). Vials of serum were stored at the JHS central repository in Minneapolis, MN, USA at −80°C until assayed. Leptin was analyzed with Human Leptin RIA kit (LINCO Research, St Charles, MI, USA) and the acceptable coefficients of variation was 10% (22). Adiponectin concentration was measured as total adiponectin by an ELISA system (R&D Systems; Minneapolis, MN, USA) (23). The inter-assay coefficient of variation was 8.8%. No biological degradation has been described using stored specimens, indicating a high validity for our measurements. We measured CRP using immunoturbidimetric CRP-Latex assay from Kamiya Biomedical Company following manufacturer’s high-sensitivity protocol. The inter-assay coefficients of variation on control samples repeated in each assay were 4.5 and 4.4% at CRP concentrations of 0.45 and 1.56 mg/L respectively. The reliability coefficient for masked quality-control replicates was 0.95 for the CRP assay.

### CVD risk factors used as covariates

Cardiovascular disease risk factor information was ascertained during the JHS Exam 1 (2000–2004) through standard procedures and questionnaires. In all participants, the clinic visit included physical examinations, anthropometry, survey of medical history, and current medication use and collection of blood and urine specimens for biological assessment. In-clinic standing height and weight were measured in lightweight examination clothing without shoes or constricting garments. We calculated BMI as weight in kilograms divided by height in meters squared (kg/m^2^). The average of two sitting blood pressure, measured at 1-min intervals after a 5-min silent rest, was used for analysis. Participants were considered current smokers if they smoked at the time of the baseline examination. Current alcohol use was defined as “yes” if they drank in the past 12 months. Lipid variables, fasting plasma glucose, and fasting insulin were measured using standard laboratory techniques. Insulin resistance was calculated using the homeostasis model assessment for insulin resistance (HOMA-IR), and used as a continuous variable. Physical activity was assessed with a physical activity survey instrument administered by interview. The questionnaire assessed four different domains of physical activity (active living, work, home and garden, and sport and exercise indexes). A total score was composed as the sum of these domains (maximum of 24), with a higher score indicating a higher level of total physical activity (24). Antihypertensive and lipid-lowering medications were assessed during the baseline exam.

### Statistical analysis

For each individual CVD risk factor, the Student unpaired *t*-test or the chi-square test was performed in order to assess the significance of the difference between participants with and without events. The relationships between logarithmically transformed leptin, logarithmically transformed adiponectin, and CVD risk factors were assessed by calculating partial correlation coefficients adjusted for age. A proportional hazard regression model was used to assess the association of either incident CHD or incident stroke as dependent variables with log-leptin or log-adiponectin [both expressed per one standard deviation (SD) increase in the log-transformed values], using the CVD risk factors as independent variables for the adjustment. The effect measure modification potential of gender and age groups (defined using the median age of 55 years) was assessed using interaction terms in the fully adjusted models. In additional analyses we queried the associations between the two adipokines and hemorrhagic stroke. We conducted also sensitivity analyses in which we excluded participants with prevalent atrial fibrillation in order to assess if the associations are confounded by preexisting heart failure determinants (more prevalent in African Americans). All computations were performed by SAS software version 9.2 (SAS^®^ Institute Inc., Cary, NC, USA).

## Results

Our study sample, composed of 2,924 women (64%) and 1,647 men, had a mean (SD) age of 54 (13) years. During a mean of 6.2 years of follow-up, 98 incident CHD and 87 incident ischemic stroke events were documented. The unadjusted mean (SD) adiponectin levels were 6.04 (4.32) μg/mL in women and 4.03 (3.14) μg/mL in men. For leptin, the levels were 37.35 (23.90) ng/mL in women and 11.03 (10.05) ng/mL in men. The characteristics of the participants according to presence or absence of the two incident conditions and by gender are presented in Table 1. Among women, there was a statistically significant difference between participants with incident CHD and those without for the majority of covariates with the exception of HDL-cholesterol and C-reactive protein. Notably, adiponectin was higher among those with incident CHD events compared with those without (Table 1). In both men and women, participants with incident stroke had higher values of systolic blood pressure/blood pressure medication but lower physical activity levels. Women with incident stroke had also higher levels of fasting plasma glucose (Table 1).

**Table 1:** **Sex-specific descriptive characteristics of the JHS participants with and without incident CHD/stroke events^a^**.

	No conditions		Incident CHD		Incident stroke	
	Women	Men	Women	Men	Women	Men
Participants (*N*)	2,889	1,602	60	38	56	31
BMI (kg/m^2^)	32.9 (7.7)	29.8 (6.2)	30.3 (5.7)^b^	29.5 (5.5)	32.6 (6.5)	29.8 (5.2)
Adiponectin (μg/mL)	6.0 (4.3)	4.0 (3.1)	7.8 (6.1)^b^	5.3 (4.3)^b^	7.0 (5.4)	5.0 (3.8)
Leptin (ng/mL)	37.3 (23.8)	10.9 (10.1)	37.5 (31.4)^b^	14.4 (10.5)^b^	41.9 (28.4)	12.6 (9.9)
HDL-C (mmol/L)	1.41 (0.37)	1.18 (0.32)	1.41 (0.39)	1.13 (0.28)	1.45 (0.42)	1.14 (0.37)
TG (mmol/L)	1.13 (0.77)	1.33 (1.36)	1.35 (0.77)^b^	1.49 (1.30)	1.35 (0.84)^b^	1.70 (1.53)
FPG (mg/mL)	98.4 (31.6)	98.7 (29.2)	114.2 (43.1)^c^	117.0 (53.2)^b^	115.5 (46.1)^c^	110.2 (50.7)
CRP (mg/L)	5.9 (8.3)	3.4 (9.9)	6.3 (11.4)	3.1 (3.3)	6.9 (11.4)	14.7 (6.0)^d^
HOMA-IR	3.8 (2.4)	3.4 (2.4)	3.4 (1.7)	3.4 (1.6)	3.8 (2.0)	3.7 (2.9)
SBP (mm Hg)	125.4 (17.9)	127.0 (17.1)	138.0 (22.9)^d^	134.7 (19.4)^b^	145.6 (28.6)^d^	140.3 (24.0)^d^
CVD events (%)	0	0	2.06	2.32	1.92	2.36
Antihypertensive medication (%)	53.1	39.3	82.8^d^	88.9^d^	76.4^c^	74.1^d^
Lipid-lowering medication (%)	11.1	9.1	20.0^b^	32.4^d^	7.6	11.5
Current smoking (%)	14.3	22.3	25.0^b^	39.5^b^	16.1	25.8^b^
Alcohol use (%)	40.5	61.1	28.3	44.7^b^	22.2^b^	54.8
Physical activity	8.4 (2.5)	8.9 (2.6)	6.7 (2.1)^d^	7.1 (2.3)^d^	7.0 (2.6)^d^	7.6 (2.7)^b^

*^a^Data are presented as mean ± SD or as percentage when appropriate*.

*^b^*p* < 0.05 for sex-specific comparison between groups*.

*^c^*p* < 0.001 for sex-specific comparison between groups*.

*^d^*p* < 0.0001 for sex-specific comparison between groups*.

*BMI indicates body mass index; HDL-C, high-density lipoprotein cholesterol; TG, triglycerides; SBP, systolic blood pressure; FPG, fasting plasma glucose; CRP, C-reactive protein; HOMA-IR, insulin resistance by homeostasis assessment model HOMA-IR; CHD, coronary heart disease; CVD, cardiovascular diseases*.

### Correlations between leptin and adiponectin and other variables

Age-adjusted correlates of log-transformed leptin and adiponectin with the CVD risk factors considered in our study are presented in Table 2. Among both men and women, adiponectin and leptin were significantly correlated with all the risk factors with the exception of systolic blood pressure. Among men, adiponectin was not significantly correlated with C-reactive protein. Leptin was highly positively correlated with BMI and HOMA-IR in both women and men (*r* = 0.66 in women and *r* = 0.73 in men, for BMI and *r* = 0.38 in women and *r* = 0.50 in men, for HOMA-IR). Adiponectin was highly positively correlated with HDL-cholesterol (*r* = 0.37 in women and *r* = 0.39 in men) and negatively correlated with HOMA-IR (*r* = −0.41 in women and *r* = −0.30 in men; Table 2).

**Table 2:** **Age-adjusted partial correlations of log-adiponectin and log-leptin to individual cardiovascular risk factors/study covariates**.

Variable	Adiponectin				Leptin			
	Women (*N* = 2,911)		Men (*N* = 1,643)		Women (*N* = 2,913)		Men (*N* = 1,639)	
	*r*	*p*-Value	*r*	*p*-Value	*r*	*p*-Value	*r*	*p*-Value
Adiponectin	–	–	–	–	−0.22	<0.0001	−0.22	<0.0001
Leptin	−0.22	<0.0001	−0.22	<0.0001	–	–	–	–
BMI	−0.23	<0.0001	−0.22	<0.0001	0.66	<0.0001	0.73	<0.0001
SBP	0.02	0.36	0.02	0.49	0.02	0.23	0.10	0.0002
HDL-C	0.36	<0.0001	0.39	<0.0001	−0.12	<0.0001	−0.29	<0.0001
TG	−0.27	<0.0001	−0.30	<0.0001	0.11	<0.0001	0.26	<0.0001
FPG	−0.25	<0.0001	−0.16	<0.0001	0.22	<0.0001	0.26	<0.0001
CRP	−0.17	<0.0001	−0.03	0.26	0.25	<0.0001	0.06	0.02
HOMA-IR	−0.40	<0.0001	−0.31	<0.0001	0.38	<0.0001	0.50	<0.0001

*HR indicates hazard ratio*.

### Multivariable-adjusted hazard regression models of leptin and adiponectin

No effect modification for gender was observed for the association of serum adiponectin and leptin with incident CHD (*p* for interaction = 0.38) or stroke (*p* for interaction = 0.77). However, we kept the analyses stratified by gender given the observed gender-differences in these associations. No effect modification for age group was observed for the association of adiponectin with incident CHD (*p* for interaction = 0.56). The results of the multivariable proportional hazard regression analyses for the gender-specific associations of log-transformed adiponectin and leptin with incident CHD and stroke are presented in Table 3. Among women, the hazard ratio (95% confidence interval) of incident stroke per one SD increase in log-adiponectin was 1.41 (1.04–1.91); *p*-value of 0.03. Notably, no association was present among men between adiponectin and incident CHD or incident stroke. No association was detected between leptin and incident CHD or stroke for neither women or men (Table 3). Due to very few hemorrhagic stroke events (*N* = 3), the association between adiponectin and hemorrhagic stroke [2.05 (0.74–5.66)] was unreliable due to a very large confidence interval. In the sensitivity analysis with exclusion of participants with prevalent atrial fibrillation, the association between adiponectin and ischemic stroke was statistically significant, 1.30 (1.01–1.67).

**Table 3:** **Associations^a^ of adiponectin and leptin with incident coronary heart disease and stroke in the Jackson Heart Study (*N* = 4,571)**.

	Incident coronary heart disease (*N* = 98)			Incident stroke (*N* = 87)		
	HR	95% C.I.	*p*-Value	HR	95% C.I.	*p*-Value
Adiponectin						
Women^b^ (*n* = 2,911)	1.32	0.99–1.77	0.06	**1.41**	**1.04–1.91**	**0.03**
Men (*n* = 1,627)	0.90	0.56–1.47	0.68	1.18	0.79–1.74	0.42
Leptin^c^						
Women (*n* = 2,913)	1.35	0.78–2.35	0.29	0.99	0.64–1.55	0.98
Men (*n* = 1,639)	1.18	0.62–2.24	0.62	0.57	0.19–1.68	0.31

*^a^Models were adjusted for age, body mass index, systolic blood pressure, blood pressure medication, HDL-cholesterol, triglycerides, C-reactive protein, insulin resistance by HOMA-IR, smoking, and physical activity*.

*^b^*p*-Value for interaction of adiponectin by gender was 0.38 for incident CHD and 0.77 for incident stroke*.

*^c^*p*-Value for interaction of leptin by gender was 0.06 for incident CHD and 0.76 for incident stroke*.

## Discussion

### Principal findings

Our study carried out in the largest community-based sample of African Americans showed that among women, but not among men, adiponectin was associated with incident ischemic stroke. No association was present between leptin and incident CHD or incident stroke. Nevertheless, adiponectin levels were significantly higher among the participants with incident CHD events compared with those without.

### In the context of previous literature

Very few previous investigations have assessed among African Americans the associations of adiponectin or leptin with incident CVD events (12, 17). In an investigation within the Cardiovascular Health Study (CHS) with 3,300 participants (that included approximately 700 African Americans), there was no association between adiponectin and incident CHD after adjusting for CVD risk factors (17). Thus, our findings of no association between adiponectin and incident CHD align with some of the previous findings.

Our results indicating a higher level of adiponectin among participants with incident CHD are intriguing but not at odds with some of the previous studies. Adiponectin is an adipose tissue-derived collagen-like protein that beneficially affects many pathways that may be relevant for the development of atherosclerosis, including glucose and lipid metabolism, inflammation, endothelial function, as well as thrombogenesis, and it is therefore supposed to protect from CHD and ischemic stroke (11). Experimental *in vitro* and animal studies have shown that adiponectin beneficially affects many pathways that may be related to the development of CVD. Administration of adiponectin in animal models improves insulin sensitivity and may have anti-atherogenic and anti-inflammatory properties (25, 26). Moreover, in some case-control studies, individuals with CHD or cerebrovascular disease have lower adiponectin levels than healthy controls (25, 26). This association generally follows a “dose-response” relationship, with lower adiponectin levels in more severe forms of CHD (27). However, results from prospective studies in humans provide inconsistent results, with only some showing significant inverse associations between adiponectin and risk of CHD (28–32). In the Health Professionals Follow-up Study, men who were free of diagnosed CVD and who were in the highest compared to the lowest quintile of adiponectin levels had a significantly 44% decreased risk of subsequent CHD over 6-year follow-up, independent of other cardiovascular risk factors (33). These observations were confirmed among diabetic men in this same cohort (28), and by an 8-year follow-up study of men reported by Frystyk et al. (29). Consistent with these findings, low plasma adiponectin levels have been shown to predict the progression of coronary calcification (34). The Strong Heart Study, including individuals of American Indian heritage with a very high prevalence of type 2 diabetes, and the male British Regional Heart Study observed inverse relationships between adiponectin and risk of CHD, associations that became nevertheless non-significant after adjustment for risk factors (30, 35). Additionally, Kuller et al. reported no significant association with risk of fatal CHD among men with the metabolic syndrome during 18 years of follow-up (36). Similarly, the MONICA/KORA study in Germany did not find a significant inverse association between adiponectin and risk of CHD (16). Moreover, a recent investigation in prospectively enrolled diabetic patients suffering with ischemic heart disease and following them up for a just over a year (37), the group with higher levels of adiponectin unexpectedly had a higher rate of a composite end point comprising cardiac and cerebral events. Similarly, among participants with higher levels of adiponectin, there was a direct association between adiponectin and incident CHD in a nested case-control within the CHS cohort (38). In the Health ABC biracial cohort, higher circulating concentrations of adiponectin were associated with higher risk of CHD in older African Americans, even accounting for CHD risk factors (12). A doubling of adiponectin was associated with a 37% higher risk of incident CHD (hazards ratio, 1.37; 95% confidence interval, 1.01–1.87) after multivariable adjustment (12). Therefore, it can be concluded that although several studies reported an inverse association between adiponectin levels and CHD there are investigations that indicated the opposite.

In terms of cerebrovascular events and the two adipokines, our finding of a direct association between higher adiponectin levels and incident stroke is intriguing. Several investigations have shown that low adiponectin levels were associated with disturbed glucose metabolism but not with the risk of ischemic stroke (4, 39–41). The data on adiponectin are nevertheless conflicting, with even large prospective studies yielding opposing results. These discrepancies are probably due to the different characteristics of the populations studied (42). Our African American population has a very high prevalence for the majority of CVD risk factors, especially BMI. This seems to be the cause of a relatively larger variance for the two adipokines studied when compared with white populations (33, 43), and might be an explanation for our findings. In a different direction, leptin appears more as a reasonable predictor of stroke, especially hemorrhagic stroke, based on the majority of the available evidence (3, 44, 45). In our previous investigation to examine the association of increased plasma leptin concentration with prevalent stroke and CHD, leptin was significantly associated with stroke only in women, and no significant association was observed in men (22). As showed in our current investigation using longitudinal data, the association did not persist. It remains to be seen, using future events, if this null association is not due to relatively few incident events available. Nonetheless, our current investigation in African Americans in JHS adds valuable information because this association of adiponectin with stroke is less assessed and it is the subject of significant controversy.

In regards to the gender-differences that we found within our study population, the information that we have for adiponectin is derived mainly from cohorts that were exclusively composed of one gender only. For example, prospective studies of the association of circulating adiponectin levels with risk of CHD have been conducted predominantly among male populations. As an exception, the British Women’s Heart and Health Study found a non-significant inverse relationship between adiponectin and CHD (35). In that study, high molecular weight (HMW) adiponectin and the HMW to total adiponectin ratio were also not significantly related to risk of CHD (46). However, the sample size in that study was limited, including only 167 cases and 333 controls. In the 20-year prospective analysis of the Rancho Bernardo Study, high plasma adiponectin levels were related to a significantly lower risk of non-fatal CHD among men only, whereas adiponectin was not significantly associated with risk of fatal CHD in either sex (15). Non-fatal events among women were not available in that report. In the KORA study, adiponectin levels were not related to risk of CHD among women (16). In a meta-analysis by Sattar et al. (47), leptin was not associated with CHD events among women enrolled in the British Women’s Heart Health Study, but significantly associated with CHD among men enrolled in the Northern Sweden investigation. In aggregate this meta-analysis found a statistically non-significant association between leptin and CHD risk that attenuated to the null following adjustment for BMI (47). Leptin was not associated with ischemic stroke among postmenopausal women recruited in the Women’s Health Initiative (48). Leptin was associated with stroke among men but not among women participants from the Northern Sweden study (4).

### Potential mechanisms

It is intriguing to speculate that an explanation for our findings of a direct association between adiponectin and incident stroke is the phenomenon of “adiponectin resistance,” which has been advanced to explain paradoxical increases in adiponectin-associated adverse outcomes (26, 49). An alternative explanation is that adiponectin is produced in response to vascular inflammation to counter the atherosclerotic process (50, 51). According to this hypothesis, adiponectin would serve as a marker of disease severity in patients with clinical CVD or older adults with subclinical CVD, but not to the same degree in younger populations free of prevalent CVD. There is also the possibility that, in addition to its protective actions, adiponectin has direct harmful effects, which could be more operative in older individuals. Indeed, adiponectin has increased energy expenditure through direct actions in the central nervous system in mice (52), an effect that, if present in humans, could be particularly deleterious in older adults by potentially accelerating sarcopenia. Notable, in our study age was not an effect modifier of the association of adiponectin with incident CHD. In a different direction, the HMW adiponectin isoform appears to be the most potent activator of insulin-sensitizing (25) and anti-atherogenic (53) pathways. This raises the possibility that the heterogeneous epidemiological findings reported thus far for total adiponectin could relate to different proportions of HMW adiponectin. We are not able at present to confirm this, as HMW adiponectin is not yet available within our cohort. On the other hand, the relatively higher prevalence of heart failure known to be present in African American women does not seem to explain the present association as similar results were obtained after excluding participants with prevalent atrial fibrillation. Further multiracial investigations should query if the association that we found is racially or gender driven.

### Implications

Despite documented preclinical cardiometabolic benefits and favorable cross-sectional clinical associations, elevated concentrations of circulating adiponectin are associated with a significantly increased risk of incident ischemic stroke among women. These findings suggest that, adiponectin has putative harmful properties. Whether in our population of African Americans with a relatively high prevalence of cardiometabolic risk factors adiponectin harbors harmful properties, or it is produced in response to vascular inflammation to counter the atherosclerotic process, or the putative “adiponectin resistance” phenomenon acts, should be further investigated.

### Strengths and limitations

The main strength of our investigation is that this study addresses important research questions using prospective CVD events data from the largest community-based sample of African Americans, the JHS, a cohort with a strict protocol of follow-up and high quality-control data. On the other hand, generalizability to other ethnic group cannot be applied. Another limitation is the relative low number of participants with incident CVD events, as incident events are accumulating.

## Conclusion

In the largest community-based sample of African Americans, there is a direct association between adiponectin and incident stroke among women but not among men, an association that deserves further investigation.

## Conflict of Interest Statement

The authors declare that the research was conducted in the absence of any commercial or financial relationships that could be construed as a potential conflict of interest.
